# Themed series in organo-fluorine chemistry

**DOI:** 10.3762/bjoc.4.11

**Published:** 2008-04-25

**Authors:** David O'Hagan

**Affiliations:** 1 School of Chemistry, University of St Andrews, Fife, KY16 9ST

It is a great pleasure to be able to introduce this Themed series on organo-fluorine chemistry in the *Beilstein Journal of Organic Chemistry*. The introduction of fluorine into organic molecules is widely practiced particularly when tuning the properties of molecules for specialist functions. Of particular prominence is the role fluorine substitution finds in pharmaceutical development [1], and selective fluorination has made a major contribution to the bioactivity of a wide range of agrochemical products [2]. Organo-fluorine compounds have also found a significant role in soft materials chemistry such as liquid crystals, photoresist polymers and self assembling monolayers [3].

Allied to this breadth of activity is a steady development in the number and range of fluorination reagents and methodologies. DAST/Deoxofluor, HF:amine reagents and TBAF have secured a central role in the armory of organic chemists and the ready availability and improvements in their formulations are allowing these reagents to be incorporated beyond the research lab and into process development. Indeed micro-reactor technology is enabling elemental fluorine to be used in large scale organo-fluorine production [4]. The introduction in the early 1990's of air stable electrophilic fluorinating reagents such as Selectfluor [5] has been revolutionary and has opened up many new methods for fluorine introduction, and provided the foundation for intense research efforts into asymmetric fluorinations leading now to some exquisite catalytic asymmetric methodologies [6].

The changes in behaviour of a molecule after the introduction of a fluorine atom continue to be unpredictable, and understanding such behaviour remains a driver in organo-fluorine research [7]. Investigations into the stereoelectronic influence of fluorine and the nature of weak interactions between fluorine and other substituents remains an active area of research and for example important insights into how fluorinated drugs interact with proteins are emerging as a result of accumulating crystallographic data of protein-drug interactions [8]. Exploring the role and nature of fluorous molecules has been an intense area of research internationally [9], one which has had a relatively short lead time since the seminal paper of Horváth and Rábai in 1994 [10]. In the area of medical imaging, positron emission tomography (PET) has undergone a step change in growth across all developing countries, with an exponential increase in the installation of PET cameras and cyclotrons to generate isotope for labelling ligands and diagnostic probes. ^18^F is an important isotope in PET because it has a relatively long half life (t_1/2_ = 110 min) and methods for introducing fluorine, appropriate to PET synthesis are in demand and will continue to grow. So the field is active and exciting and it is in that context that the *BJOC* feels it appropriate to profile a themed series in the area. Contributions to the series come from an international grouping of noted experts in fluorine chemistry and we are delighted that they have agreed to contribute their papers for such a successful launch.
